# A Novel Intelligent Fault Diagnosis Method of Rolling Bearings Based on the ConvNeXt Network with Improved DenseBlock

**DOI:** 10.3390/s24247909

**Published:** 2024-12-11

**Authors:** Jiahao Song, Xiaobo Nie, Chuang Wu, Naiwei Zheng

**Affiliations:** College of Mechanical Engineering, Inner Mongolia University of Technology, Hohhot 010051, China

**Keywords:** ConvNeXt, DenseNet, fault diagnosis, CWT, rolling bearing

## Abstract

Rolling bearings are critical rotating components in machinery and equipment; they are essential for the normal operation of such systems. Consequently, there is a pressing need for a highly efficient, applicable, and reliable method for bearing fault diagnosis. Currently, one-dimensional data-driven fault diagnosis methods, which rely on one-dimensional data, represent a mainstream approach in this field. However, these methods exhibit weak diagnostic capabilities in noisy environments and when confronted with insufficient sample sizes. In order to solve these limitations, a new fault diagnosis method for rolling bearings is proposed, which combines the ConvNeXt network and improved DenseBlock into a parallel network with a feature fusion function. The network can fully extract the global feature and the detail feature of the signal and integrate them, which shows a good diagnostic ability in the face of a strong noise environment. Additionally, the Dy-ReLU function is introduced into the network, which enhances the generalization ability of the network and improves the convergence speed. Comparative experiments show that this method still has strong fault diagnosis capability under the condition of noise pollution and insufficient training samples.

## 1. Introduction

With the continuous progress and development of science and technology, especially the popularization and development of automation technology, the mainstream development direction in the field of machinery has begun to shift to smart manufacturing. The rolling bearing is one of the most important rolling components of the machinery, and its good working condition is the key factor in ensuring that the equipment can operate normally [1,2,3,4]. Since the beginning of the 1960s, researchers have been developing mechanical equipment operation condition monitoring and fault diagnosis technologies, which have gradually formed a relatively perfect, emerging, comprehensive engineering discipline.

With the proposal of deep learning methods, which has attracted many scholars’ attention, deep learning has been widely applied in the field of machinery and equipment fault diagnosis because it can mimic human thinking and learning activities, enabling it to have a high learning ability. Deep learning benefits from a fast learning speed, especially when processing large amounts of data [5,6,7,8]. Some research results have already been reported. For example, Zhang, S. et al. [9] integrated the channel-space attention mechanism into the deep residual network, which improved the diagnostic accuracy of the network. Lv, Z.H. et al. [10] devised a fault diagnosis method using active learning-deep neural networks (AL-DNNs) and domain adversarial networks (DANNs). Zhou, K. et al. [11] introduced a semi-supervised fault diagnostic approach for automotive gearboxes with nine faults by designing a semi-supervised deep convolutional generative adversarial network (DCGAN). In order to address different conditional label distributions, Yang, B. et al. [12] presented a deep targeted migration learning (DTTL) method, which counters the traditional migration learning assumption that all target domains are unlabeled and existing methods share the same conditional label distribution.

Yan, X.A. et al. [13] tackled the fault diagnosis under variable operating conditions for rolling bearings by proposing a deep-order wavelet convolutional variational self-encoder (DOWCVAE), which enhances diagnosis accuracy by extracting potential feature information layer-by-layer from augmented-order spectral data. Deng, Y.F. et al. [14] addressed the challenging open-set diagnostic transfer problem in deep migration learning (OSDT) by designing a theory-guided progressive transfer learning network (TPTLN). Ruan, D.W. et al. [15] innovatively designed a CNN with rectangular input shapes and rectangular convolution kernels; the kernel size is determined according to the length of the signal at different attenuation ratios. Due to the uncertain working conditions of train bogie bearings, the traditional fault diagnosis method under fixed working conditions cannot obtain accurate results, so a fault diagnosis method for variable working conditions is needed. Wei, Z.X. et al. [16] proposed a third-order tensor model alongside a density-based affinity propagation tensor (DAP-Tensor) clustering algorithm, achieving accurate fault diagnosis in unsupervised conditions.

To solve the issue of bearing signal fluctuations due to operating conditions, Zhao, X.L. et al. [17] developed a multi-scale deep graph convolutional network (MS-DGCN), which ensures high diagnostic accuracy and generalization. Xiao, Y.M. et al. [18] combined Bayesian variational learning with Transformer technology to construct a probabilistic Bayesian–Transformer model for reliable RMFD, verifying its effectiveness and generalizability under three working conditions. Zhang, J.S. et al. [19] addressed the imbalance between fault samples and healthy samples in real engineering environments by proposing an improved denoising self-encoder (DAE) method based on the bottleneck layer self-attention mechanism (MDAE-SAMB), achieving effective fault diagnosis in the case of a small sample number.

Chen, B.Y. et al. [20] proposed the product envelope spectrum optimization gram (PESOgram) method. The method has a great ability to identify the fault characteristic frequencies of bearings, which results in superior diagnostic accuracy. Lastly, Lin, J. et al. [21] tackled the challenge of extracting generalized diagnostic knowledge in meta-learning methods applied to fault diagnosis by developing a generalized model-independent meta-learning (GMAML) for small-sample rolling bearing fault diagnosis under various operating conditions.

Deep learning methods have been extensively applied by the aforementioned research to the fault diagnosis of various devices and components, achieving remarkable results. Nevertheless, there are certain limitations to these methods. For instance, Transformer-based fault diagnosis methods often struggle with inadequate sample sizes; traditional CNN-based diagnostic methods can suffer from issues such as gradient vanishing or explosion in deep networks; and shallow networks may fail to extract features comprehensively.

DenseNet was proposed by Huang, G. et al. [22] in 2017. First, we know that in traditional CNNs, L layers have L connections. But in DenseNet, the connection number is calculated as L(L + 1)/2, and the input of each layer is derived by the outputs of all preceding layers. This structure effectively mitigates the gradient vanishing problem. Additionally, each layer in DenseNet receives inputs from all the preceding layers and shares its feature mappings with all the successive layers, promoting extensive feature reuse and improving feature transmission through the network. Consequently, DenseNet is widely utilized in fault diagnosis because of its distinctive advantages. Li, Y.H. et al. [23] introduced a deep reinforcement learning (DRL) method based on advantage actor-critic (A2C), achieving high diagnostic accuracy by using DenseNet as the policy and value network for A2C agents. In the field of small-sample as well as zero-sample fault diagnosis, Zhang, Y.M. et al. [24] combined DenseNet with generalized zero-sample learning (GZSL) to enhance fault diagnosis accuracy for high-speed trains with insufficient data on bogie composite faults. Jiang, G.J. et al. [25] used a capsule neural network (CN) that has a fast routing algorithm in combination with an improved DenseBlock, which effectively alleviates the problem of long training time and high requirement of training equipment for capsule networks. Additionally, they further proposed an adaptive dynamic activation convolutional capsule network [26] (ADAC-CN) that combines a convolutional layer with a pooling layer, which makes the network gain greater ability to extract deep features while reducing the parameter number. Then, they introduced a dynamic ReLU activation function to the ADAC-CN, which makes the network extract the feature more efficiently. Wang, C.D. et al. [27] proposed an efficient fault diagnosis network based on a refined prototype and correlation weighting Manhattan distance, which incorporates a multi-scale feature extraction (MSFE) module and a sparse nonlocal attention (SNLA) module and plays a good role in targeting the problem of the bearing box fault diagnosis in the case of lacking samples. Yang, J.L. et al. [28] proposed the BCMPN by targeting the multivariability of the working conditions of bearings and the unevenness of the valid sample number. This network uses a multi-scale mask preprocessing mechanism and Brownian distance covariance, which gains a high diagnostic accuracy level in cross-domain fault diagnosis as well as zero-sample fault diagnosis. Xu, Z. et al. [29] proposed a Vision Transformer (ViT) model that utilizes multi-information fusion and can perform efficient bearing fault diagnosis with small numbers of data samples. Peng, C. et al. [30] proposed a conditional depth convolution countermeasure generation network (C-DCGAN), which achieves efficient data augmentation, effectively expands the small sample data, and improves the fault classification.

ConvNeXt, proposed by Liu, Z. et al. [31] in 2022, integrates the successful designs of ResNet and Swin Transformer to achieve smoother network gradients, leading to faster convergence and improved network performance. ConvNeXt has demonstrated excellent results in image classification tasks, though its application in fault diagnosis remains limited. Yang, S.W. et al. [32] converted harmonic drive vibration signals into symmetric dot-matrix images and used ConvNeXt to classify features under various operating conditions, thereby validating ConvNeXt’s effectiveness and accuracy in fault diagnosis problems. Zang, C. et al. [33] enhanced ConvNeXt with digital twin and transfer learning theories, utilizing a similar attention module and college channel attention network to improve ConvNeXt for diagnosing and analyzing rolling bearings, confirming its efficacy and achieving high diagnostic accuracy. However, Zang, C. et al. [33] did not investigate the diagnostic capacity of the network in the face of strong noise data.

Appellate research has applied the ConvNeXt network method to fault diagnosis research of different devices and components and achieved excellent results at the same time. However, the following problems still exist:

- In the actual working environment, the operating environment of the bearing is quite complex, and the vibration signal of the bearing will inevitably be polluted by noise, which will lead to fault characteristics that are difficult to identify and make the fault diagnosis work difficult. However, noise pollution has not been considered in most of the existing studies.
- In the actual working environment, it is very difficult to obtain sufficient and effective sample data, but most existing studies have not simulated the situation of insufficient samples.
- The environment in engineering practice is not static, so the diagnostic method needs to have good generalization ability and stability. At present, most studies are limited to the same dataset, and validation on multiple datasets is not considered.

Here in this paper, we combined the improved DenseBlock with the ConvNeXt network, proposing a new deep network for fault diagnosis of rolling bearings. Initially, the method processes bearing vibration signals collected by sensors using continuous wavelet transform (CWT) to generate diagnostic samples. Then, the DenseNet with improved Denseblock and the ConvNeXt network are combined into a parallel network, the global and detailed features of the signal are extracted at the same time, and the DY-ReLU activation function is combined to enhance the network training with minimal computational cost. The reliability of this method is validated through experiments using the CWRU rolling bearing dataset and the HIT aero-engine inter-axle bearing failure dataset, with comparisons to other methods. The experimental results show that the DCN method achieves the highest accuracy in the diagnostic experiments for four working conditions and four noise environments for the CWRU rolling bearing dataset, and in the diagnostic experiments for the HIT aero-engine inter-axle bearing failure dataset, the DCN method proves the robustness of the method with a stable and excellent performance. Meanwhile, the stability test of the DCN method is carried out by considering the reduction in samples and simulating the insufficient number of samples. The results show that the method has excellent diagnostic capability and can provide stable diagnostic capability in the case of an insufficient number of samples. Finally, through ablation experiments, it is verified that the dynamic ReLU function can significantly improve the feature extraction ability of the network and improve the diagnostic accuracy with a small computational cost. The main work of this paper is summarized as follows:

- A continuous wavelet transform is used to fully extract the deep information of the signal and realize the conversion from a one-dimensional signal to a two-dimensional time-frequency image.
- A new two-branch parallel network is constructed that uses a DenseNet branch and a ConvNeXt branch with an improved Denseblock to extract global features and detailed features of images, respectively.
- The Double-Way Fusion Block is introduced to perform channel attention processing on the features extracted from the DenseNet branch and ConvNeXt branch before fusion, so as to complement the information of the two branches and obtain a more comprehensive feature extraction effect.
- The traditional static ReLU function is replaced with the dynamic ReLU activation function, which gives the network a better generalization ability, an enhanced network expression ability, and a better convergence speed.

## 2. Model Construction

### 2.1. ConvNeXt Network

**Figure 1:**
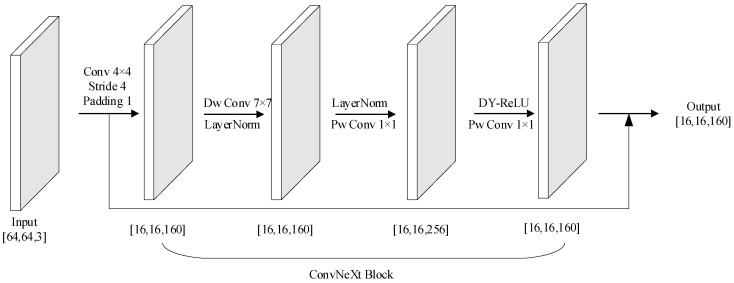
Schematic diagram of the ConvNeXt network structure.

ConvNeXt is a set of convolutional neural networks with improved design. It starts from the original ResNet and gradually improves the model by borrowing the design of Swin Transformer, which finally results in a set of architectures that provide excellent performance as well as scalability and simplicity, as shown in Figure 1.

**Figure 2:**
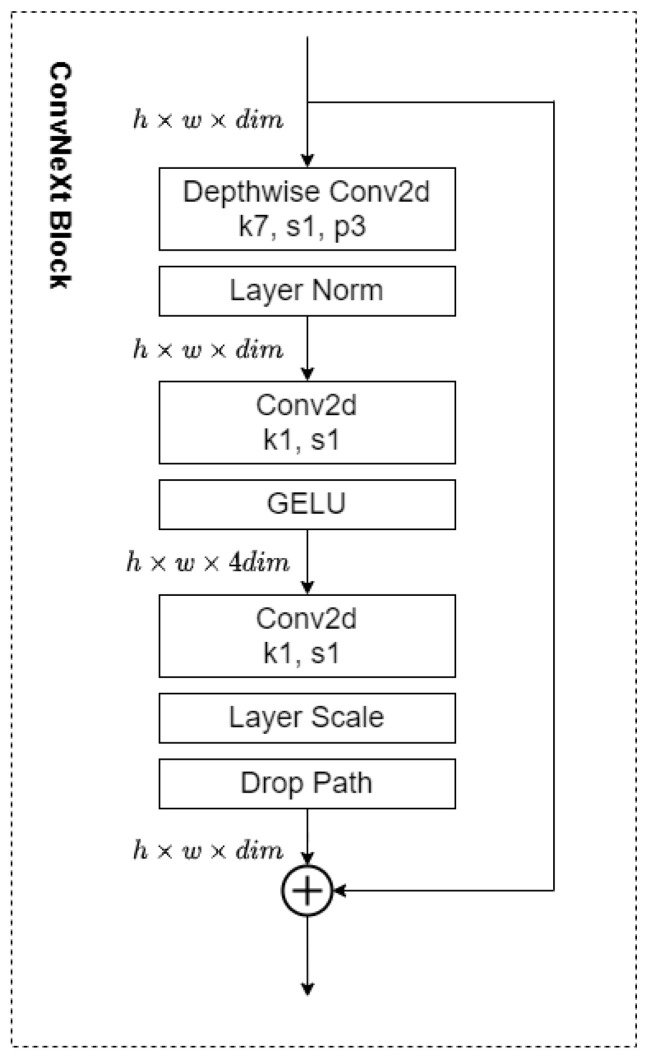
Schematic diagram of the ConvNeXt Block structure.

The input layer of this network is followed by an initial convolutional layer, which typically contains a larger convolutional kernel (7 × 7 or 4 × 4) for the initial extraction of image features. The part that follows the initial convolutional layer is an important constituent structure of the ConvNeXt network called the ConvNeXt Block, which borrows some key ideas from traditional convolutional neural networks and Transformer networks in its design. A standard ConvNeXt Block structure is shown in Figure 2.

It can be seen that in the ConvNeXt Block, the data output from the initial convolutional layer goes into the Depthwise Convolution (DW) convolutional layer for further convolutional operations, and this convolution method is beneficial in increasing the depth of the network. Additionally, it can also significantly increase the amount of computation and enhance the model’s ability to capture the details by processing them channel by channel. Moreover, in the ConvNeXt Block, the commonly used Rectified Linear Unit (ReLU) activation function is replaced by the smoother Gaussian Error Linear Unit (GELU), there are fewer activation functions than in other networks, and the LayerNorm (LN) is used instead of the BatchNorm (BN). The combination of these improvements allows ConvNeXt to outperform most neural networks. Higher accuracy could also be achieved simultaneously.

### 2.2. Improved DenseBlock

When the number of layers of a CNN becomes progressively deeper, the path to the output layer from the input layer becomes correspondingly longer. Thus, the problem of vanishing gradients occurs. Therefore, DenseNet proposes a simple and effective method: all the layers in front of the neural network are directly linked to the layers behind it, i.e., the layer’s output is linked to all the layers that are behind it.

For example, we have an input image P_0, then the formula of input–output in a conventional CNN is shown as Equation (1):

(1) $$P_{l}=H_{l}\left(P_{l-1}\right)$$

For DenseNet, its input–output equation at layer l is shown in Equation (2):

(2) $$P_{l}=H_{l}\left(\left[P_{0},P_{1},\ldots ,P_{l-1}\right]\right)$$

where $H_{l}\left(\cdot \right)$ denotes the nonlinear combinatorial function, which typically includes BN, ReLU, pooling, and Conv operations. $P_{l}$ denotes the layer l output.

**Figure 3:**
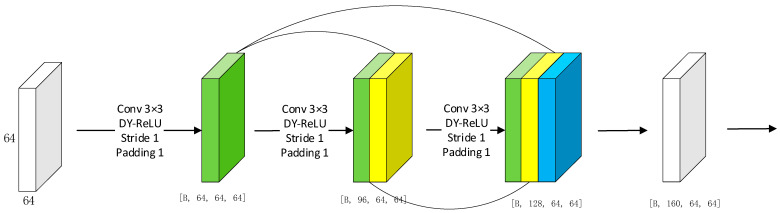
DenseBlock structure.

DenseBlock is a fundamental module of the DenseNet neural network, known for its stronger gradient mobility. This characteristic effectively mitigates the gradient vanishing problem, enhances feature propagation, preserves low-dimensional features, and significantly reduces the number of parameters [22]. DenseNet reuses feature information from previous layers during the training process. Regardless of the network’s depth, features from earlier layers are uniformly transferred to later layers. However, this characteristic of DenseBlock limits its ability to fully utilize all feature information. To improve the network and solve these problems, we made some improvements to DenseBlock [34] so the block can adjust the number of output feature maps. This adjustment reduces the reliance on features from earlier layers and increases the importance of features from later layers. Assuming each layer of DenseBlock produces *k* feature maps, the number of feature maps produced by the lth layer is determined by Equation (3).

(3) $$k_{l}=\beta k\times \frac{l}{n}$$

where *n* denotes the number of layers in DenseBlock and β is a hyperparameter chosen empirically in the range of 2 to 6. We can see from Equation (3) that the closer the layer is to the output layer, the greater the number of filters it contains, and therefore the greater the effect on the output. In this paper, the use of the improved DenseBlock as the feature extraction layer makes the ConvNeXt network incorporate the feature reuse function of DenseNet, which improves the accuracy and computational efficiency of the network. The DenseBlock structure is shown in Figure 3.

### 2.3. Dynamic Activation Function

We describe Dynamic ReLU (DY-ReLU) in this section. Unlike a series of static functions such as ReLU, the parameters of DY-ReLU are generated from hyperfunctions on all input elements. DY-ReLU neither increases the depth nor the width of the network, but due to the powerful expressiveness of the DY-ReLU function, it can effectively increase the model capacity with barely any additional computational cost [35].

**Figure 4:**
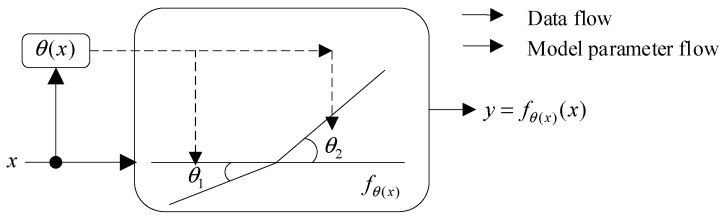
DY-ReLU.

DY-ReLU can dynamically adjust the slope of the segmented linear function by input. The principle of DY-ReLU is shown in Figure 4. $\theta \left(x\right)$, which indicates the hyper function, and $y=f_{\theta \left(x\right)}\left(x\right)$, which indicates the activation function, are two parts of DY-ReLU. The parameters of the segmentation function $y=f_{\theta \left(x\right)}\left(x\right)$ are calculated from the hyper function $\theta \left(x\right)$. Every input element $x=\left\{x_{c}\right\}$ transmits all the information it contains into the hyper function $\theta \left(x\right)$ to activate the channel.

Assuming x is the input of the network, the conventional static ReLU is denoted as y=max {x,0}, and the activation function $y_{c}=max\;\{x_{c},0\}$ is the $c^{th}$ channel, with an input of $x=\left\{x_{c}\right\}$. The ReLU can be regarded as the parametric segmented linear function $y_{c}=max_{k}\left\{a_{c}^{k}x_{c}+b_{c}^{k}\right\}$, whereas the DY-ReLU allows the static segmented linear function to be extended by adjusting $a_{c}^{k}$ and $b_{c}^{k}$ through the inputs $x=\left\{x_{c}\right\}$, and thus the parameters of $y_{c}=max_{k}\left\{a_{c}^{k}x_{c}+b_{c}^{k}\right\}$ can be expressed as:

(4) $$y_{c}=f_{\theta (x)}\left(x_{c}\right)=\max_{1\leq k\leq K}\left\{a_{c}^{k}(x)x_{c}+b_{c}^{k}(x)\right\}$$

**Figure 5:**
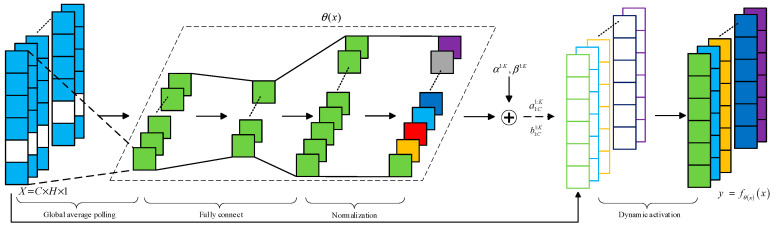
Structure of DY-ReLU.

The structure of DY-ReLU is shown in Figure 5. For an input tensor $x=\left\{x_{c}\right\}$, its input dimension is assumed to be C×H×1. Firstly, a global average pooling operation is used to compress its spatial information, and the compressed information is assumed to be ν. ν is then spread by a fully connected layer, and its dimension is reduced from C to C/R, where R is the hyperparameter. The final output is shown in Equations (5) and (6) as follows:

(5) $$a_{c}^{k}(x)=\alpha^{k}+\lambda_{a}\Delta a_{c}^{k}(x)$$

(6) $$b_{c}^{k}(x)=\beta^{k}+\lambda_{b}\Delta b_{c}^{k}(x)$$

where $\alpha_{k}$, $\beta_{k}$, $\lambda_{a}$, $\lambda_{b}$ are the hyperparameters. $\alpha_{k}$ and $\beta_{k}$ are the initial values of $a_{c}^{k}$ and $b_{c}^{k}$, respectively. $\lambda_{a}$ and $\lambda_{b}$ are scalars controlling the range of residuals with default values of 1.0 and 0.5, respectively.

### 2.4. Continuous Wavelet Transform

**Figure 6:**
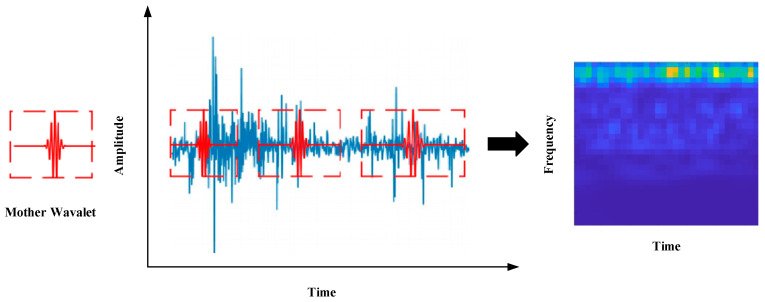
Schematic diagram of the continuous wavelet transform.

Continuous wavelet transform [36] is a widely used technique for signal data analysis, which analyzes the signal by wavelet function. In addition, CWT can generate two-dimensional time–frequency maps by using one-dimensional vibration signals. The basic equation of the continuous wavelet transform is shown in Equation (7).

(7) $$W_{f}(a,b)=\frac{1}{\sqrt{\left|a\right|}}\int_{-\infty }^{\infty }f(t)\psi {\left(\frac{t-b}{a}\right)}^{*}dt$$

f(t) denotes the original time domain signal, the wavelet function is denoted by ψ(•), and $\psi^{*}(•)$ denotes the complex conjugate of the function ψ(•). The scale parameter a controls the expansion and contraction of the wavelet function. The displacement parameter b controls how the wavelet function moves along the time axis. The operation process of the continuous wavelet transform is shown in Figure 6.

### 2.5. Multi-Feature Fusion Module

**Figure 7:**
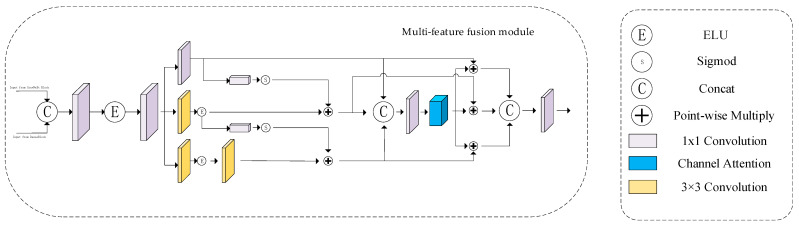
DFB.

The main structure of the Double-Way Fusion Block [37] (DFB) is shown in Figure 7. In the method proposed in this paper, after the input of training samples, the DenseBlock branch and the ConvNeXt Block branch are processed, respectively. The DenseBlock branch is mainly used to extract local features of the image and pay attention to the detailed features of the image. The ConvNeXt Block branch mainly extracts the global features of the image, utilizes the advantages of the deep separable convolution in ConvNeXt, extracts the global information in the image, and compensates the information of the DenseBlock branch through the subsequent DFB module. Finally, all the local and global features extracted from the two branches are put into the DFB for fusion; the information from the input DFB from the two branches is fully scrambled and mixed, and the combined information is input into the subsequent network for processing.

(8) $$f_{i}=DFB(Dense(f_{i-1}),ConvNeXt(f_{i-1}))$$

As can be seen from Formula (8), the level *i* feature *f_i_* is obtained by fusing the feature *f_i_*_−1_ output by the previous DFB module after being processed by the DenseBlock and ConvNeXt Block.

## 3. Proposed Method

### 3.1. Fault Diagnosis Process

**Figure 8:**
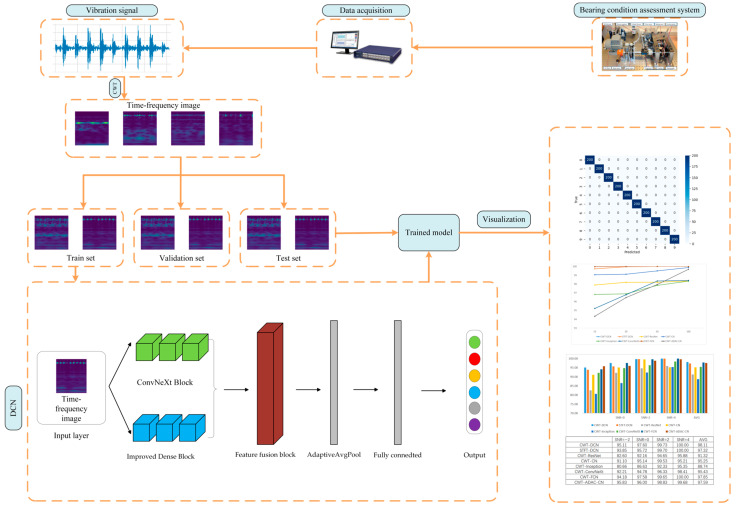
DCN-based fault diagnosis process.

In this paper, the basic flow of the proposed diagnosis method is shown in Figure 8. The specific steps of the proposed method are as follows:

Step 1: First, install sensors on the mechanical equipment that need to collect bearing vibration signals. Then, obtain the vibration signals when the bearings are working through the collection system.

Step 2: Classify and select the acquired signals to ensure that the acquired samples are real, effective, and sufficient.

Step 3: Perform CWT processing on the signals and generate two-dimensional time–frequency diagrams by using the vibration signals collected in Step 1.

Step 4: Divide the converted time–frequency map into the training set, validation set, and test set according to a certain ratio.

Step 5: Train the network using the training set divided in Step 4, and save the best model of the network in the whole training process.

Step 6: Use the test set to test the optimal network model in Step 5 and obtain the final result.

### 3.2. Construction of the DCN Model

**Figure 9:**
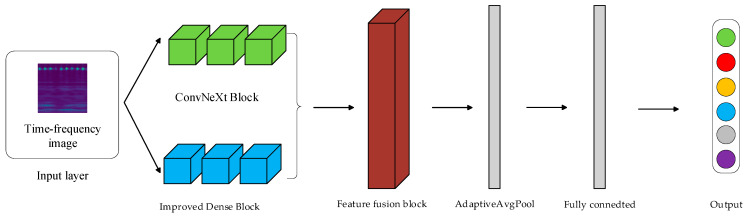
Structure of the DCN.

The DCN network is mainly composed of an improved DenseBlock and ConvNeXt Block, and the network diagram is shown in Figure 9. After the input of the training samples, they are directly processed by the DenseBlock branch and the ConvNeXt Block branch, respectively, and the feature maps after the processing of the two branches are input into the subsequent Dual-Feature Fusion Block.

After the multi-feature fusion module, the data are fed into the classifier composed of normalization, DY-ReLU, Adaptive Mean Pooling, a flatten layer, and a linear layer, and the final classification operation is completed through the classifier.

## 4. Experiment and Result Analysis

In order to test the validity and generality of the proposed method, the rolling bearing dataset of Case Western Reserve University [38] and the intermediate bearing failure dataset of the aero-engine at Harbin Institute of Technology [39] are selected for validation in this paper. We used Pytorch2.5.1 with cuda12.4 to build the model, and a workstation configured with Intel^®^ Xeon^®^ Gold 5118 CPU (Intel, Santa Clara, CA, USA) with NVIDIA^®^ Quadro RTX™ 6000 GPU (NVIDIA, Santa Clara, CA, USA) was used for training. During the training phase, we used the Adam optimization algorithm, with the batch_size at 32, the epoch set to 50, and an initial learning rate of 0.001, which decays when there is no growth in accuracy for 10 consecutive rounds of training.

In order to make the comparison results more objective, this paper compares DCN with several other methods to fully verify the reliability of DCN. The chosen methods are widely used and have been verified countless times in the field of fault diagnosis. The following is a brief introduction to these methods:

(1) ResNet: ResNet was proposed in 2015 by He et al. [40]. ResNet greatly improves the solution to the degradation problem of deep networks with its residual connectivity property while significantly reducing the number of parameters.
(2) CN: CapsNet (CN) was proposed by Sabour et al. [41] in 2017. As the information of features in CN is in the form of vectors, the network is able to retain the relative positional relationships between the input object parts, i.e., the network has a built-in understanding of 3D space. Compared to traditional CNNs, CN requires only a small amount of data to achieve good learning results.
(3) Inception: This network was proposed by Szegedy et al. [42] in 2015. The core structure of Inception is the Inception layer, and the data input to this layer will be passed in parallel to multiple convolutional and pooling operations, which eventually merge their outputs.
(4) TST: TST is based on the architecture proposed by A. Vaswan et al. [43] in 2017, improving its attention module to accommodate time series data. The method is designed to better capture temporal dependencies in a time series using the Transformer self-attention mechanism and positional coding and has become a popular method in the field of time series analysis.
(5) ConvNeXt: ConvNeXt was proposed by Liu, Z. et al. [31] in 2022. ConvNext incorporates the successful designs of ResNet and Swin Transformer to achieve smoother network gradients, which leads to faster convergence and further increases the performance of the network.
(6) FCN: This network was proposed by Jiang, G.J. et al. [25] in 2024, and it combines a capsule neural network (CN) with a fast routing algorithm with an improved DenseBlock, which effectively mitigates the problems of long training time and high requirement of training equipment for capsule networks.
(7) ADAC-CN: The network was proposed by Jiang, G.J. et al. [26] in 2024. It combines the convolutional layer and pooling layer into one layer, enabling the network to extract deeper features while reducing the parameter number. In addition, they introduced dynamic ReLU into the ADAC-CN, which further improves the efficiency of the feature extraction and achieves a higher accuracy in the cross-domain diagnostics of bearings.

### 4.1. Case 1

#### 4.1.1. Datasets and Data Preprocessing

**Figure 10:**
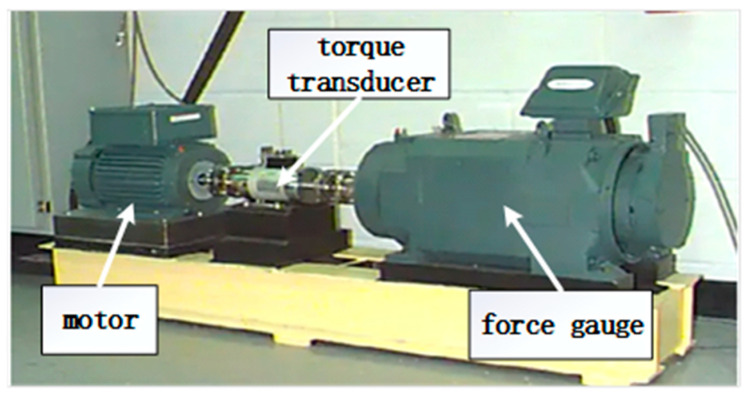
CWRU rolling bearing data acquisition test bed.

The rolling bearing dataset from the Case Western Reserve University [38] (CWRU) Rolling Bearing Data Centre was selected for validation in this case, and the CWRU rolling bearing data acquisition test bed is shown in Figure 10.

In the CWRU dataset, there are four types of faults based on rotational speed, and each broad category contains four fault types, which are normal, inner-ring faults, outer-ring faults, and rolling-body faults. The faults have different damage levels of 0.007 inches, 0.014 inches, 0.021 inches, and 0.028 inches, respectively. Since the data with a damage level of 0.028 inches contain only the inner ring failure and the rolling body failure, the data with a damage level of 0.028 inches were not selected for this part of the test. The working conditions for this dataset are shown in Table 1, and the failure types are shown in Table 2.

**Figure 11:**
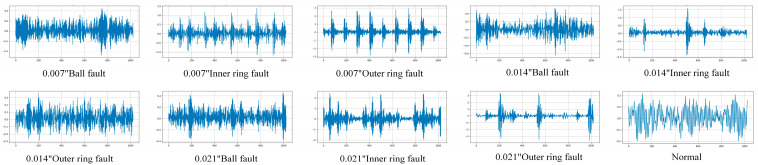
Original vibration signal.

**Figure 12:**
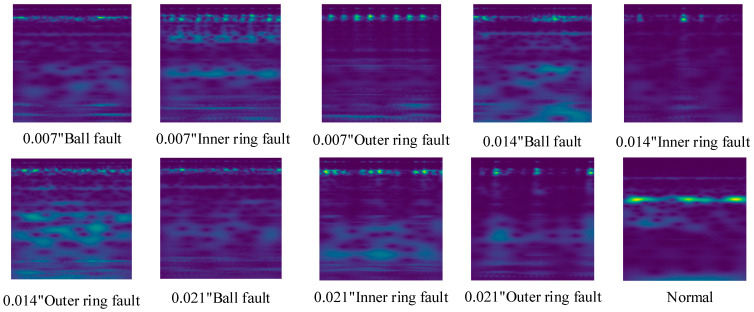
Time–frequency diagram of a vibration signal.

**Figure 13:**
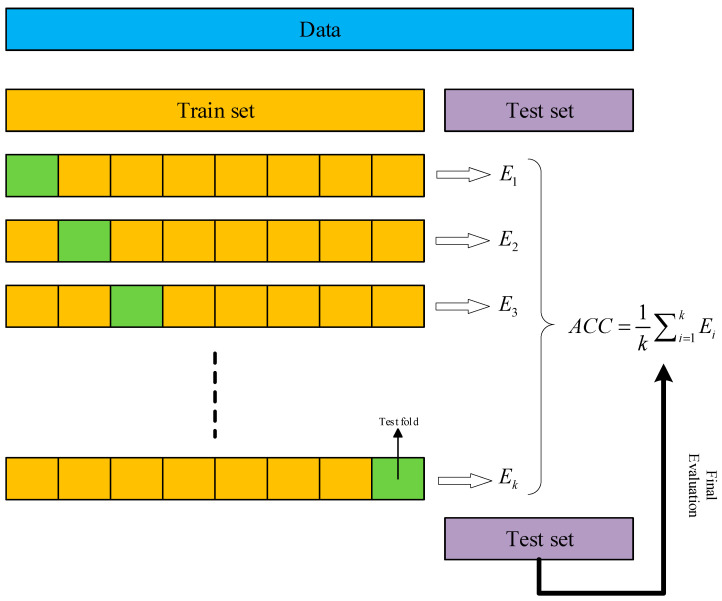
Schematic diagram of the k-fold cross-validation approach.

In order to obtain the information in the time series more comprehensively and to obtain enough samples, we used overlapping sampling during the acquisition process to generate the samples. First, create a 1024-data length sliding window with a step size of 200. Then collect 500 samples for each kind of fault, respectively, so there are a total of 5000 samples being generated. Subsequently, perform CWT processing on the signals to generate two-dimensional time–frequency diagrams. Figure 11 shows the 10 vibration signal images of a bearing under 1797 RPM, and Figure 12 shows the time–frequency image generated from the samples by continuous wavelet transform. In order to avoid data leakage during training and to make sure that the training results are real and reliable, this paper chooses the k-fold cross-validation method for model training, and the schematic diagram of the validation method is shown in Figure 13. In the construction of the dataset, 200 samples from each fault sample are reserved as the test set, and these samples are not contained in the training and validation sets; it is a new and unknown dataset for the model, which can effectively assess the generalization ability of the model.

From Figure 11 and Figure 12, it can be seen that the signals of several faults are relatively similar; for example, the 0.007″ ball fault vs. the 0.014″ ball fault and the 0.014″ inner ring fault vs. the 0.021″ inner ring fault. These similar signals make it easy to extract similar features during feature extraction, which brings some difficulties to the subsequent classification.

#### 4.1.2. Experimental Results and Analysis

(1) Fault Diagnosis in a Simulated Noise Environment

In actual engineering environments, noise interference exists between mechanical equipment and between components of the same equipment, resulting in unavoidable noise contamination of the acquired signals when signal acquisition is performed on rolling bearings. Therefore, it is necessary to verify whether the method can diagnose correctly in a noisy environment.

In Section 4.1.1, we mentioned that a total of 5000 samples were collected. In this section, we added Gaussian noise with different signal-to-noise ratios (SNRs) to these samples in order to simulate noise pollution in a real environment. The formula for the SNR is shown in Equation (9).

(9) $$SNR=10\log\frac{P_{signal}}{P_{noise}}$$

where $P_{signal}$ denotes the power of the original signal, and $P_{noise}$ is the power of the added noise.

**Figure 14:**
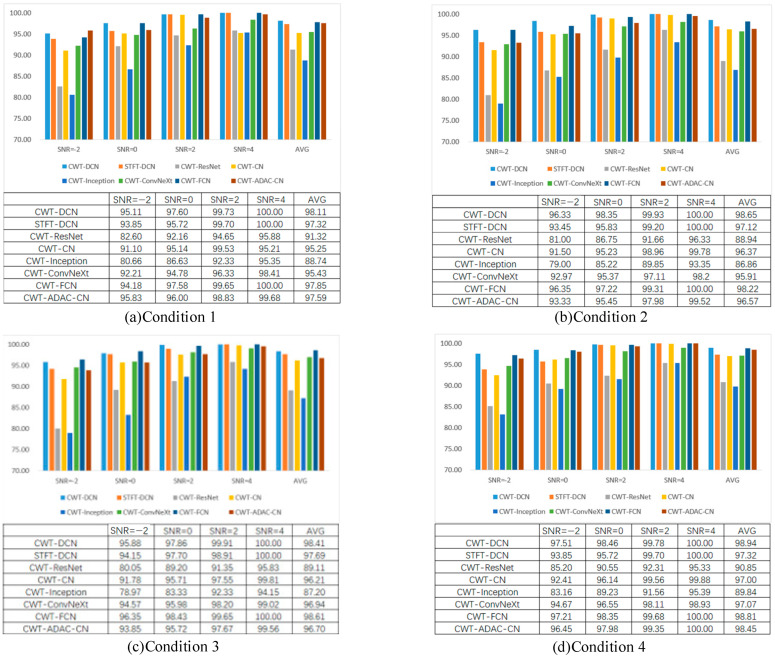
Diagnostic accuracy in a noisy environment.

In order to avoid the influence of random events and make the experimental results more reliable, we chose to use the data of the four working conditions in Table 1 to carry out simulation experiments. Each kind of experiment was carried out 10 times, and the final results were taken as the average of 10 experiments. The results of this part of the experiment are shown in Figure 14.

As can be seen in Figure 14, when SNR = 4 and SNR = 2, the diagnostic accuracies of the various methods are maintained at a high level, which indicates that these methods have good diagnostic ability in the case of weak noise. When SNR = 0, the accuracy of various methods starts to show a substantial decrease; only DCN and CN have a smaller decrease in accuracy and stable performance, indicating that DCN integrates the advantages of DenseNet and the ConvNeXt network, which is capable of effective extraction of deeper features and can maintain a stable diagnostic ability under stronger noise pollution. When SNR = −2, the power of the noise has already exceeded the power of the original vibration signal at this time, in which case only the DCN method still maintains high diagnostic accuracy; the accuracy of CWT-DCN is higher than STFT-DCN due to the fact that the CWT has a certain advantage compared to STFT in dealing with non-smooth signals and is able to analyze the signals on different scales.

Combining the information shown in the above icons, the proposed method shows better diagnostic ability than the other comparative methods under four different working conditions and different noise situations. It indicates that the proposed method has good noise resistance.

**Figure 15:**
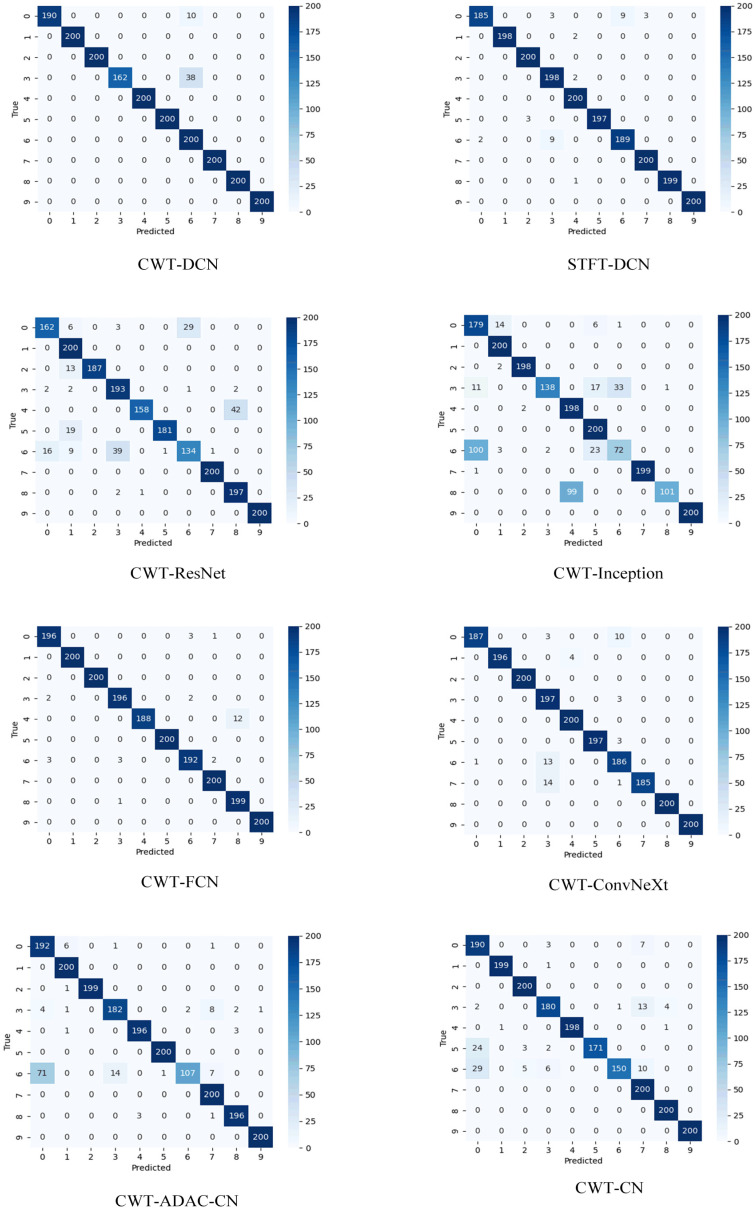
Confusion matrices.

In order to further understand the classification details of various methods, we output the test results in the form of confusion matrices, as shown in Figure 15. This part shows the confusion matrix picture of the classification results in the case of Case 3 with SNR = 0, where the horizontal coordinate is the predicted labels, and the vertical coordinate is the real labels.

From Figure 14, it can be seen that CWT-DCN is able to classify most of the faults perfectly, and misclassification occurs in only two fault types. This part of the results clearly demonstrates the excellent ability of CWT-DCN.

(2) Fault diagnosis in case of insufficient simulation samples

In the actual engineering environment, data collection is often difficult, and due to various natural factors, the data may fluctuate at certain moments, so this part of the data cannot be used for fault diagnosis, which often makes us unable to obtain enough data for diagnosis. Therefore, it becomes especially important to simulate the case of insufficient samples and verify the diagnostic ability of the model in these circumstances.

**Figure 16:**
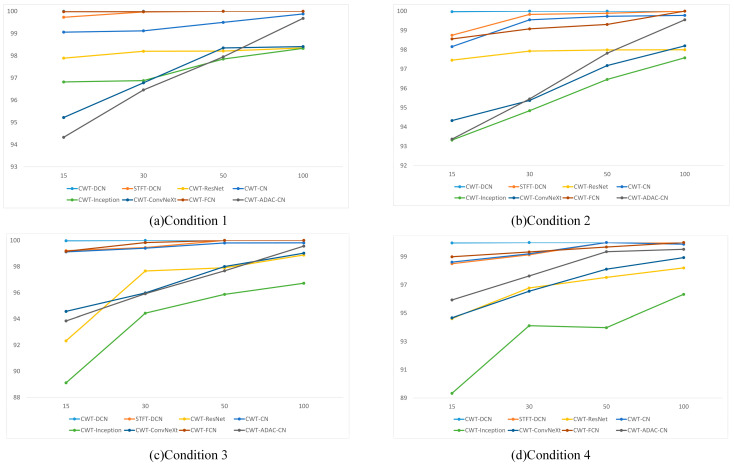
Diagnostic accuracy for different sample sizes.

In this section, we still use the four working conditions in Table 1: manually divide the training samples for each fault, choosing 100 samples, 50 samples, 30 samples, and 15 samples, respectively. The validation and test sets are the same as in part (1). Figure 16 shows the diagnostic accuracy of each method with a different number of samples.

As can be seen from Figure 16, in the case of the 50 and 100 sample sizes, all methods achieve their respective standards, and the diagnostic accuracies are in line with the normal performance of each method. When the sample size is reduced to 30, the accuracy of each method begins to show a decline in different sizes, and only CWT-DCN, STFT-DCN, and CWT-CN are stable. When the sample size is only 15, the diagnostic accuracy of the methods other than CWT-DCN and STFT-DCN shows a significant decrease, which indicates that DCN can extract features more comprehensively and ensure diagnostic accuracy even when the sample sizes are not sufficient.

### 4.2. Case 2

#### 4.2.1. Datasets and Data Preprocessing

**Figure 17:**
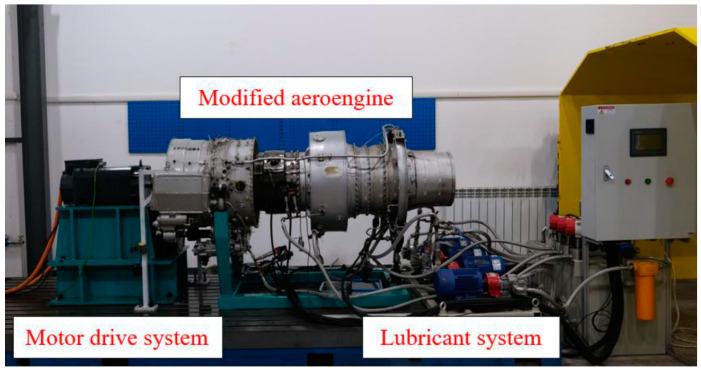
Aero-engine test bed.

The dataset of the aero-engine inter-axle bearing failures from the School of Aerospace at Harbin Institute of Technology (HIT) was selected for validation in this case, and the HIT aero-engine test bed is shown in Figure 17.

**Figure 18:**
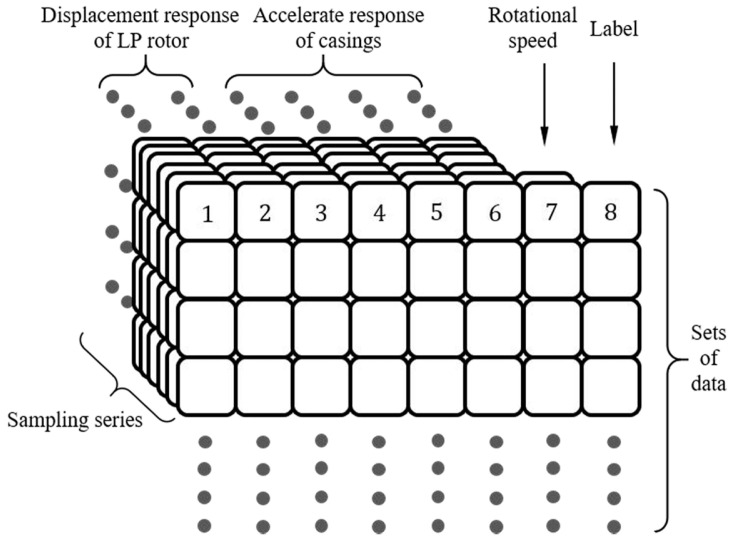
Schematic representation of the dataset.

The dataset includes three types of conditions: inner ring fault, normal state, and outer ring fault. The inner ring fault is further categorized into two cases: one with a failure depth of 0.5 mm and length of 0.5 mm and another with a failure depth of 0.5 mm and length of 1.0 mm. The aero-engine in the dataset features two structures: a low-pressure rotor and a high-pressure rotor, each operating at different rotational speeds. The low-pressure rotor operates between 1000 and 5000 r/min, while the high-pressure rotor operates between 1200 and 6000 r/min. The speed ratios between the low- and high-pressure rotors range from 1.2 to 1.8, and the specific structure of the data is shown in Figure 18. The specific rotational speeds and the speeds of the low-pressure rotor and high-pressure rotor are shown in Table 3, and the fault types and labels are shown in Table 4.

**Figure 19:**
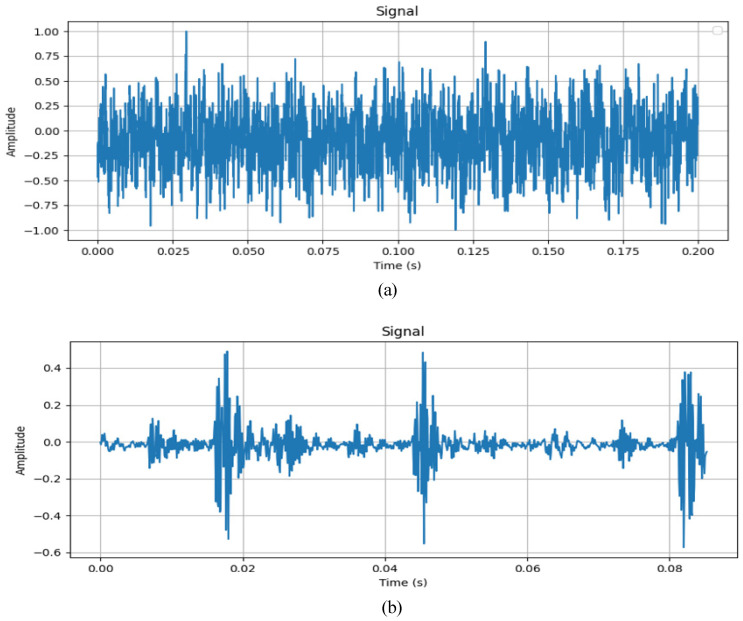
Original vibration signal. (**a**) HIT dataset and (**b**) CWRU dataset.

As can be seen in Figure 19, the waveform of the data contained in the HIT dataset is similar to a random signal; however, by observing the waveform of the CWRU dataset, it can be found that the waveform of this dataset is an obvious impact waveform. This is because of the structure of the aero-engine; the sensor cannot be mounted closer to the faulty bearing when collecting data. As a result, the signal of the bearing will be attenuated when it is transmitted, and the collision between the structures of the aero-engine will generate noise, which makes the vibration signal messy and difficult to distinguish. In this case, the fault diagnosis method should have a certain anti-interference ability and be able to effectively extract the fault characteristics in order to achieve high diagnostic accuracy. Therefore, it is more realistic to use this dataset to verify the effectiveness of the method.

In the HIT dataset, the same overlapping sampling method as in Case 1 was used to sample the data with an LP speed of 3000 r/min, HP speed of 3600 r/min, and speed ratio of 1.2. A total of 300 samples were taken as the test set, 100 for the validation set, and 400 for the training set.

#### 4.2.2. Experimental Results and Analysis

In the HIT aero-engine intershaft bearing failure dataset, multiple sensors are employed to monitor the vibration signals of the bearings. When these sensors monitor the same target, a consistency check of their signals is essential to eliminate inaccuracies, inconsistencies, or missing measurements caused by environmental factors. The reliability of the sensor data is equally crucial. Measurement uncertainties and sensing errors can arise from manufacturing inaccuracies, environmental changes, and other factors. These factors all have an impact on the monitoring data. If these factors are ignored, they can significantly reduce prediction accuracy.

In this paper, we used variance to evaluate the reliability of sensor data. By assessing the variance, the study aims to identify and address the inconsistencies and uncertainties in the sensor data to enhance the accuracy of fault diagnosis. Higher variance denotes more volatility and lower reliability of the signal. Conversely, lower variance denotes a higher reliability of the signal. According to the variance data of each sensor, each sensor is assigned a certain weight, and the weighted fusion data can be obtained.

**Figure 20:**
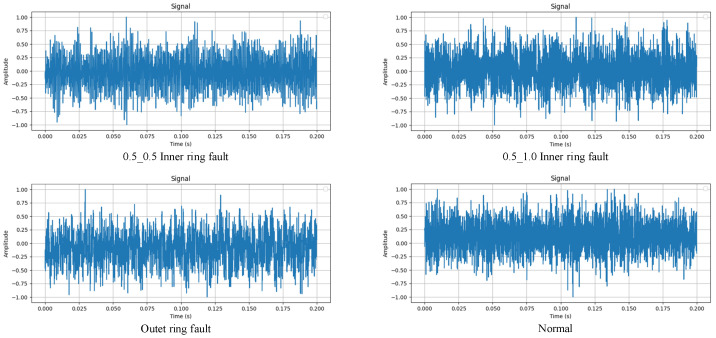
Weighted fusion signal.

As can be seen in Figure 20, the waveforms of the data contained in the HIT dataset are closer to random signals, and the special waveforms of the various faults are not obvious, making diagnosis difficult.

**Figure 21:**
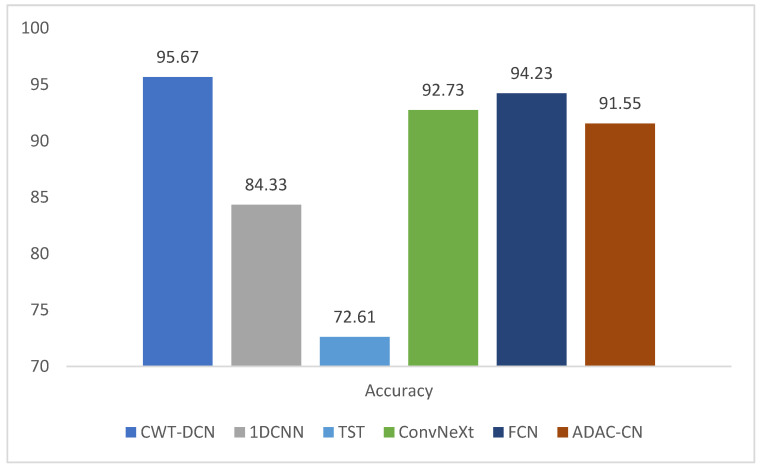
Diagnostic accuracy by method.

**Figure 22:**
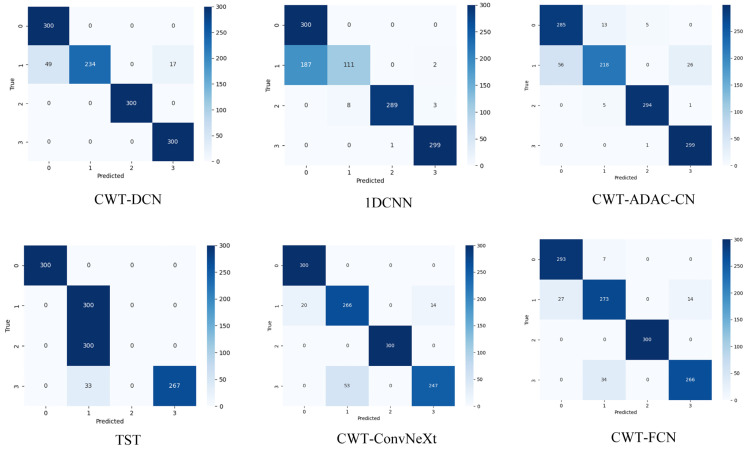
Confusion matrices.

To verify the diagnostic capability of the proposed method for this dataset, 1DCNN and TST are used to compare with CWT-DCN in this section. The diagnostic accuracy of each method at an LP speed of 3000 r/min, HP speed of 3600 r/min, and speed ratio of 1.2 is shown in Figure 21, and the confusion matrices are shown in Figure 22.

**Figure 23:**
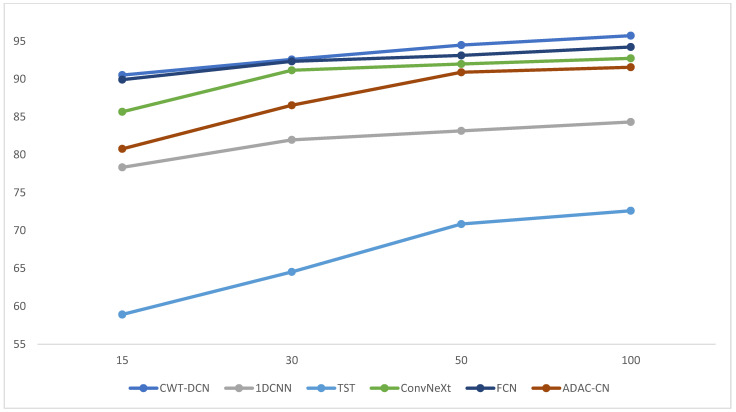
Diagnostic accuracy in the case of insufficient samples.

Similarly, the section also tested the above five methods in the same simulated sample insufficiency case as in Case 1, aiming to check whether the methods can maintain high accuracy in an environment closer to the actual working conditions. The diagnostic accuracy in the case of simulated sample insufficiency is shown in Figure 23.

As can be seen in Figure 23, the diagnostic accuracy of the method using time–frequency plots as input samples is significantly better than the method using one-dimensional time-series information as input samples, indicating that the features embedded in the signals can be more comprehensively demonstrated after CWT processing. In addition, when the sample size is smaller than 50, the accuracy of TST shows a substantial decrease, which indicates that the empirical models represented by TST need a large amount of data support and may not be appropriate for use when the amount of data is insufficient. Taken together, these two points prove that the proposed method has good generalization and applicability, is able to cope with most of the practical engineering application scenarios, and can make a reliable diagnosis.

### 4.3. Ablation Experiment

#### 4.3.1. Activation Function

This part of the ablation test aims to explore the effect of the activation function on model training accuracy and training efficiency. In the DCN method, the dynamic activation function DY-ReLU is introduced, which enables the network to adaptively activate and capture feature information. In addition, DY-ReLU can improve the training effect of the network with barely any additional computational cost. In order to verify the ability of DY-ReLU to improve the efficiency and accuracy of model training, in this ablation test, the data of Case 1 are used for training. To minimize the influence of random events, the test was repeated 10 times, and the final results were averaged. The results of the ablation test are shown in Table 5.

As can be seen in Table 5, after ablation tests using DY-ReLU, ReLU, and GELU as activation functions in the DCN method, the DCN method has a different number of parameters, diagnostic accuracy, and time used for each training batch. Among them, when the method uses DY-ReLU as the activation function, the number of network parameters is not very different, and the training time is similar compared to the ReLU and GELU activation functions, but after using DY-ReLU as the activation function, the accuracy is significantly improved compared to the other activation functions, which indicates that the DY-ReLU activation function can be activated adaptively, and the features are extracted more efficiently.

#### 4.3.2. DenseBlock

This part of the ablation experiment aims to explore the effect of a traditional DenseBlock and an improved DenseBlock on the model. As can be seen from Table 6, there is little difference in training time between the two DenseBlocks, but there is a big difference in diagnostic accuracy, which indicates that the improved DenseBlock fully considers the important difference between feature graphs and plays a role in removing redundant features and reusing features.

#### 4.3.3. Hyperparameters

This part of ablation experiment aims to study the effect of convolution kernel hyperparameters on the model.In this part, we set different sizes of convolution kernels in DenseBlock and ConvNeXt Block, respectively, where DenseBlock is 2 × 2, 3 × 3, and 5 × 5, and ConvNeXt Block is 3 × 3, 7 × 7, and 10 × 10. The training time and diagnostic accuracy were the criteria for the final results.

As can be seen from Table 7 and Table 8, the convolution kernel size of the DenseNet branch and ConvNeXt branch is selected to be 3 × 3 and 7 × 7, respectively, at 10 epochs, which can make the network achieve a balance in terms of training time and diagnostic accuracy. It is worth noting that the neural network did not converge completely at 10 epochs, so the data recorded in Table 8 do not reflect the real performance of the network because the partial ablation experiment was designed to explore the effect of the convolution kernel size on the training accuracy, and the network with incomplete convergence helps to amplify the effect of the variables.

## 5. Conclusions

In this paper, a DCN-based rolling bearing diagnostic method was proposed, which first performs CWT processing on the bearing vibration signals captured by the sensors to generate the samples required for diagnosis. Subsequently, the improved DenseBlock is paralleled with the ConvNeXt network, and the DY-ReLU function is introduced. Finally, we verified the method by using the CWRU rolling bearing dataset and the HIT aero-engine intershaft bearing failure dataset. Then, we compared the proposed method with other methods. The results show that the accuracy of the proposed method for Case 2 is 89.33%, 91.67%, 93.72%, and 94.50%, respectively, which is better than other comparative methods when facing the small-sample fault diagnosis cases with sample numbers of 15, 30, 50, and 100 in a strong noise environment. The accuracy of the proposed method is also better than other comparative methods for the four small-sample diagnostic cases in Case 1.

Although the method has good fault diagnosis capability in the case of noise pollution and insufficient training samples, it still cannot achieve unsupervised learning and requires manual labeling of samples, which increases the overall cost. In addition, the requirement for computing resources is still high. In the experiment, we noticed that the model occupies about 8 GB of video memory. Reducing the batch_size or the size of the input feature map should reduce the memory footprint to some extent, but we did not have enough time to explore the effects of these measures on the speed and accuracy of model training. In addition, the real-time performance of the method is low because the method requires close to 50 iterations to converge, and the training time is close to half an hour, which is too long for the actual engineering environment. Knowledge distillation or pre-training networks might be a good solution. Therefore, in future studies, this scheme will be considered to achieve low-cost and efficient unsupervised learning.

## Figures and Tables

**Table 1:** Introduction of working conditions.

Condition	Speed	Load(HP)
1	1797	0
2	1772	1
3	1750	2
4	1730	3

**Table 2:** Description of fault types.

Degree of Damage (inches)	0.007	0.007	0.007	0.014	0.014	0.014	0.021	0.021	0.021	0
Failure position	Ball fault	Inner ring fault	Outer ring fault	Ball fault	Inner ring fault	Outer ring fault	Ball fault	Inner ring fault	Outer ring fault	Normal state
Label	0	1	2	3	4	5	6	7	8	9

**Table 3:** Rotor speed and speed ratios.

LP Speed (r/min)	HP Speed (r/min)	Speed Ratio	LP Speed (r/min)	HP Speed (r/min)	Speed Ratio
1000	1200	1.2	4400	5280	1.2
1500	1800	1.2	4500	5400	1.2
2000	2400	1.2	4600	5520	1.2
2500	3000	1.2	4700	5640	1.2
3000	3600	1.2	4800	5760	1.2
3500	4200	1.2	4900	5880	1.2
3600	4320	1.2	5000	6000	1.2
3700	4440	1.2	3000	3600	1.2
3800	4560	1.2	3000	3900	1.3
3900	4680	1.2	3000	4200	1.4
4000	4800	1.2	3000	4500	1.5
4100	4920	1.2	3000	4800	1.6
4200	5040	1.2	3000	5100	1.7
4300	5160	1.2	3000	5400	1.8

**Table 4:** Fault types and labels.

Label	Failure Position	Depth_length of Damage (mm)	Speed Ratio
0	Normal	0_0	1.2
1	Inner ring	0.5_0.5	1.2
2	Inner ring	0.5_1.0	1.2
3	Outer ring	0.5_0.5	1.2

**Table 5:** Results of the ablation experiment.

Methods	Parameter Number	Accuracy	Total Time	10 Epoch Accuracy
DCN-DY-ReLU	1.854 M	100%	247.52 s	97.2%
DCN-ReLU	1.756 M	98.6%	230.68 s	95.57%
DCN-GELU	1.757 M	99.1%	233.52 s	95.64%

**Table 6:** Results of different DenseBlocks.

DenseBlock	Accuracy	Total Time
Improved	100%	270.13 s
Traditional	98.67%	265.42 s

**Table 7:** Results of time usage (10 epochs).

C\D	2 × 2	3 × 3	5 × 5
3 × 3	271.29 s	267.94 s	256.29 s
7 × 7	249.83 s	247.55 s	242.13 s
10 × 10	259.94 s	260.65 s	266.52 s

**Table 8:** Results of diagnostic accuracy (10 epochs).

C\D	2 × 2	3 × 3	5 × 5
3 × 3	90.5%	94.6%	93.7%
7 × 7	93.4%	96.8%	96%
10 × 10	92.7%	95.5%	94.5%

## Data Availability

The data in Case 1 are openly available in the Bearing Data Center at https://engineering.case.edu/bearingdatacenter/download-data-file (accessed on 15 June 2024) [38], reference number https://doi.org/10.1016/j.ymssp.2015.04.021. The data in Case 2 are publicly available on GitHub at https://github.com/HouLeiHIT/HIT-dataset (accessed on 3 July 2024) [39], reference number https://doi.org/10.37965/jdmd.2023.314.
